# Posttransplant Hyponatremia Predicts Graft Failure and Mortality in Kidney Transplantation Recipients: A Multicenter Cohort Study in Korea

**DOI:** 10.1371/journal.pone.0156050

**Published:** 2016-05-23

**Authors:** Seung Seok Han, Miyeun Han, Jae Yoon Park, Jung Nam An, Seokwoo Park, Su-Kil Park, Duck-Jong Han, Ki Young Na, Yun Kyu Oh, Chun Soo Lim, Yon Su Kim, Young Hoon Kim, Jung Pyo Lee

**Affiliations:** 1 Department of Internal Medicine, Seoul National University College of Medicine, Seoul, Korea; 2 Department of Internal Medicine, Seoul National University Boramae Medical Center, Seoul, Korea; 3 Department of Critical Care Medicine, Seoul National University Boramae Medical Center, Seoul, Korea; 4 Department of Internal Medicine, Asan Medical Center and University of Ulsan College of Medicine, Seoul, Korea; 5 Department of Surgery, Asan Medical Center and University of Ulsan College of Medicine, Seoul, Korea; 6 Department of Internal Medicine, Seoul National University Bundang Hospital, Seongnam, Korea; 7 Kidney Research Institute, Seoul National University College of Medicine, Seoul, Korea; George Washington University School of Medicine and Health Sciences, UNITED STATES

## Abstract

Although hyponatremia is related to poorer outcomes in several clinical settings, its significance remains unresolved in kidney transplantation. Data on 1,786 patients who received kidney transplantations between January 2000 and December 2011 were analyzed. The patients were divided into two groups according to the corrected sodium values for serum glucose 3 months after their transplantations (<135 mmol/L vs. ≥135 mmol/L). Subsequently, the hazard ratios (HRs) for biopsy-proven acute rejection, graft failure, and all-cause mortality were calculated after adjustments for several immunological and non-immunological covariates. 4.0% of patients had hyponatremia. Patients with hyponatremia had higher risks for graft failure and all-cause mortality than did the counterpart normonatremia group; the adjusted HRs for graft failure and mortality were 3.21 (1.47–6.99) and 3.03 (1.21–7.54), respectively. These relationships remained consistent irrespective of heart function. However, hyponatremia was not associated with the risk of acute rejection. The present study addressed the association between hyponatremia and graft and patient outcomes in kidney transplant recipients. Based on the study results, our recommendation is to monitor serum sodium levels after kidney transplantations.

## Introduction

The annual rate of kidney transplantation has steadily increased due to its proven survival benefit over other treatment options [1]. More specifically, the short-term outcomes of kidney transplant recipients have improved over the past 2 decades through the introduction of several immunosuppressive agents [2], but this improvement has not led to the improvement of long-term outcomes. Both immunological and non-immunological factors are important to predict and improve this long-term graft outcome [3], including age, human leukocyte antigen (HLA) matching, HLA immunization, ethnic background, and cardiovascular comorbidities. However, further data on possible factors related to the outcomes are needed to monitor the recipients with an efficient and personalized approach.

Hyponatremia is regarded as an important risk factor for high morbidity and mortality in several clinical settings [4]. This relationship is plausible because hyponatremia increases adverse consequences through several effects, such as decreased brain function, compromised cardiac contractility, increased insulin resistance, and abnormalities in neuromuscular function [5–8]. Additionally, hyponatremia can be induced throughout the presence of several comorbidities [9]. The clinical importance of hyponatremia has been known in the transplant field, although this issue was particularly restricted in the liver [10–12]; patients with hyponatremia exhibited low graft survival and high mortality after liver transplantation. However, there have been no studies on the relationship between hyponatremia and outcomes after kidney transplantation.

Herein, we first addressed the predictability of sodium levels on the graft and mortality outcomes in a large cohort who received kidney transplantations. Furthermore, its predictability remained significant independent of several immunological and non-immunological factors, all of which have been monitored in current clinical practice. The results indicate the necessity of adding serum sodium to the current monitoring system for kidney transplantations.

## Methods

### Ethics statement

The study protocol complies with the Declaration of Helsinki and received full approval from the institutional review board at the Seoul National University Hospital (no. 1406-125-591). The requirement of informed consent was waived by the board.

### Participants and data collection

Data on patients receiving kidney transplantation were obtained retrospectively from a cohort that consisted of patients from 2 tertiary referral centers (Seoul National University Hospital and Asan Medical Center). A total of 2,885 patients consecutively underwent kidney transplantations from January 2000 to December 2011. Patients were excluded from the analysis if they were younger than 20 years old (n = 181) or if they had received multiple organ transplantations [i.e., liver-kidney (n = 22), pancreas-kidney (n = 85), and heart-kidney (n = 4)], because these patients had a distinctive graft or patient survival rate compared with others. We obtained sodium levels 3 months after kidney transplantation with following reasons; we attempted to include patients with stable sodium statuses and exclude patients with potentially unstable statuses in the early period of transplantation. In particular, pre-transplant sodium levels were not considered because sodium levels are affected significantly by end-stage renal disease or dialysis [13]. With this rationale in mind, 3-month sodium data were available for 1,857 patients. We additionally excluded patients who had hypernatremia (>145 mmol/L) (n = 45) and had graft failure within 3 months (n = 26). Consequently, 1,786 patients were analyzed for the present study.

The clinical parameters recorded included the following: age, sex, weight, smoking, hypertension, diabetes mellitus, history of cardiovascular disease, liver cirrhosis, history of tuberculosis, origin of end-stage renal disease, previous transplantation, donor type, ABO-incompatible transplantation, and the use of medications that have hyponatremia risk, such as thiazides, selective serotonin reuptake inhibitors, and anti-psychotic drugs. In addition to the serum sodium levels, the following laboratory data were considered: potassium, chloride, bicarbonate, hepatitis B virus surface antigen, anti-hepatitis C virus antibody, HLA mismatch, and donor-specific antibody. All the laboratory parameters were obtained when the patients were stable based on the medical reviews. Corrected sodium values for serum glucose were used in the study analyses, based on the following formula: corrected sodium = measured sodium + 0.016 × (serum glucose– 100) [14]. The estimated glomerular filtration rate was calculated by using the Modification of Diet in Renal Disease equation [15]. Steroids, calcineurin inhibitors, and inhibitors of purine synthesis were used as the basic immunosuppressive agents; thus, we evaluated whether patients received tacrolimus or mycophenolate mofetil. For the primary analysis, data on the biopsy-proven acute rejection, graft failure, and all-cause mortality after 3 months of transplantation were collected. All patients were followed until graft failure or December 2014.

### Statistical analysis

The data are presented as the means ± standard deviations for the continuous variables and as the proportions for the categorical variables. The chi-squared test and Student’s *t*-test were used to compare the categorical variables and continuous variables, respectively. We divided the patients into the following two groups: normonatremia (135–145 mmol/L) and hyponatremia (<135 mmol/L). Survival curves were drawn using the Kaplan-Meier method. To compare the survival curves between the groups, the log-rank test was initially applied. To calculate the hazard ratios (HRs) of the outcomes, the Cox proportional hazard model was used, with and without the adjustments for the covariates. In the multivariate models, age, sex, estimated glomerular filtration rate, and covariates with *P*<0.1 in the univariate analysis were initially adjusted, and then all the covariates were adjusted. A *P* value less than 0.05 was considered significant. All analyses and calculations were performed using SPSS software (version 21.0, IBM Corp., Chicago, IL, USA).

## Results

### Baseline characteristics of the study subjects

**Fig 1:**
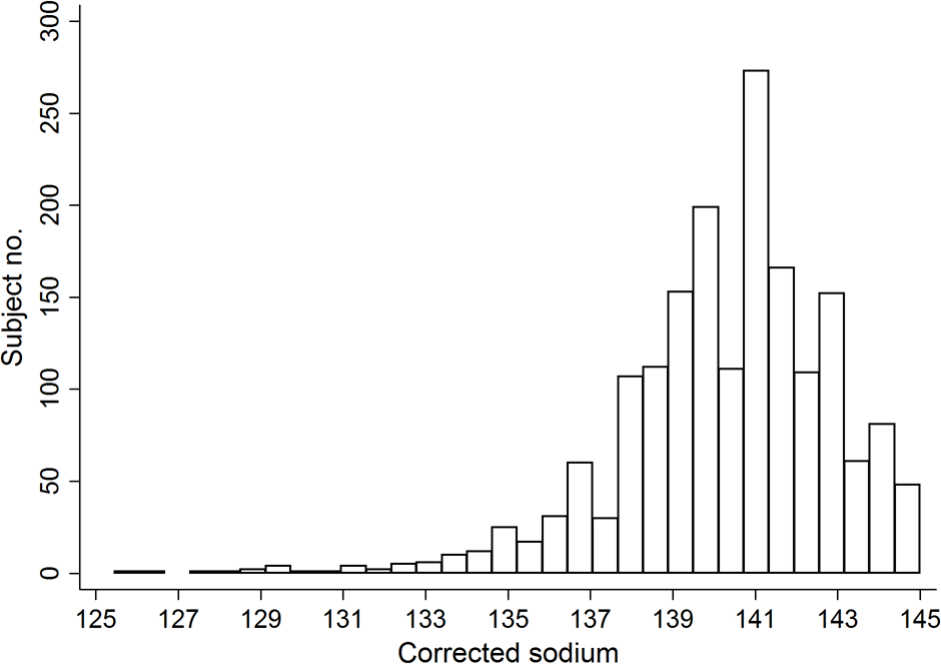
A histogram of the corrected sodium levels.

For the 1,786 subjects, the mean age was 43 years (Table 1). Among them, 4.0% had hyponatremia. The medians (interquartile ranges) of sodium and corrected sodium were 133 mmol/L (131–134) and 133.9 (131.8–134.7), respectively. The ranges of corrected sodium were from 125.5 to 134.9. Fig 1 shows the histogram for the corrected sodium. The baseline characteristics of the patients were compared between hyponatremia and normonatremia groups. Patients with hyponatremia were more likely to have diabetes as comorbidity or as a cause of end-stage renal disease, had more histories of cardiovascular diseases, and were less likely to use the induction therapy than the patients with normonatremia. The electrolyte levels, including sodium, chloride, and bicarbonate, were different between two groups. Patients were followed for the mean duration of 68 months (maximum 15 years).

**Table 1:** Baseline characteristics of the study subjects.

Parameters	Total (n = 1,786)	Normonatremia (n = 1,715)	Hyponatremia (n = 71)	*P*
Age (years)	42.9 ± 11.17	42.8 ± 11.09	45.5 ± 12.7	0.052
Male (%)	60.8	60.8	62.0	0.837
Weight (kg)	61.1 ± 10.77	61.2 ± 10.82	59.1 ± 9.20	0.112
Smoking (%)	23.4	23.7	19.7	0.454
Hypertension (%)	86.3	86.5	81.7	0.251
Diabetes mellitus (%)	20.2	19.8	29.6	0.045
History of cardiovascular disease (%)	7.3	7.0	15.5	0.007
Liver cirrhosis (%)	1.1	1.1	0	0.373
History of tuberculosis (%)	6.2	6.2	5.6	0.851
Hepatitis B virus surface antigen (%)	5.2	5.0	9.9	0.067
Anti-hepatitis C virus antibody (%)	1.3	1.3	1.4	0.961
Origin of end-stage renal disease (%)				0.003
Diabetes mellitus	15.1	14.8	21.1	
Glomerulonephritis	21.6	22.0	9.9	
Others	26.5	26.9	16.9	
Unknown	36.8	36.2	52.1	
Previous transplantation (%)	3.5	3.4	5.6	0.310
Donor type (%)				0.311
Living related	50.4	50.4	50.7	
Living unrelated	26.8	27.1	20.3	
Cadaveric	22.8	22.6	29.0	
HLA mismatch	3.2 ± 1.61	3.2 ± 1.62	3.2 ± 1.39	0.878
Donor-specific antibody (%)	1.5	1.5	0	0.296
ABO-incompatible transplantation (%)	5.0	5.1	2.8	0.382
Induction (%)	72.3	72.8	60.6	0.024
Tacrolimus (%)	58.9	59.1	53.5	0.347
Mycophenolate mofetil (%)	85.4	85.4	87.3	0.646
Thiazide (%)	1.3	1.2	0.1	0.244
Selective serotonin reuptake inhibitor (%)	0.7	0.7	0.1	0.491
Anti-psychotic drugs (%)	0.8	0.8	0	0.445
Serum sodium (mmol/L)	140.1 ± 2.79	140.4 ± 2.28	132.3 ± 2.79	<0.001
Corrected serum sodium	140.3 ± 2.59	140.6 ± 2.11	133.0 ± 2.28	<0.001
Serum potassium (mmol/L)	4.3 ± 0.52	4.3 ± 0.51	4.5 ± 0.67	0.054
Serum chloride (mmol/L)	105.9 ± 3.71	106.1 ± 3.53	101.0 ± 4.45	<0.001
Serum bicarbonate (mmol/L)	24.5 ± 3.30	24.6 ± 3.26	22.1 ± 3.37	<0.001
Serum creatinine (mg/dL)	1.2 ± 0.48	1.2 ± 0.46	1.3 ± 0.90	0.259
Estimated GFR (ml/min/1.73 m^2^)	71.6 ± 24.69	71.6 ± 24.53	70.8 ± 28.6	0.791
Follow-up duration (months)	59.6 (37.4–92.4)	59.2 (37.4–91.8)	62.7 (36.6–109.7)	0.403

HLA, human leukocyte antigen; GFR, glomerular filtration rate.

### Risk of outcomes according to the presence of hyponatremia

**Fig 2:**
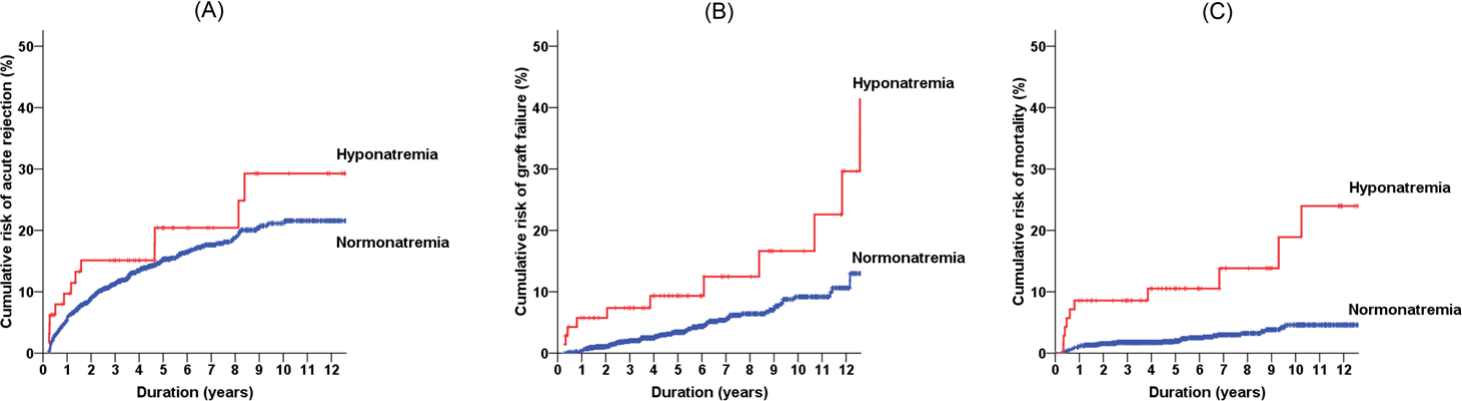
Kaplan-Meier curves for the cumulative risks of acute rejection (A), graft failure (B), and all-cause mortality (C).

During the follow-up period, 53 (3.0%) patients died and 87 (4.9%) patients had graft loss. One hundred sixty-three patients (9.1%) had at least one episode of acute rejection in the 3 months after the kidney transplantation. The causes of death were as follows: infection (n = 21), cancer (n = 11), cardiovascular disease (n = 3), and others. The graft failures were attributable to acute rejection (n = 9), chronic rejection (n = 16), recurrence (n = 5), infection (n = 8), or non-compliance (n = 4); but most other cases could not clearly determined the cause of graft failure. Fig 2 shows the Kaplan-Meier risk curves of acute rejection (A), graft failure (B), and all-cause mortality (C). The risk difference was significant in the outcome of graft failure and mortality (*P*s were <0.001 by the log-rank test), but the difference was not significant with acute rejection (*P* = 0.251). When the most common two causes for mortality (i.e., infection and cancer) were only considered, the infectious mortality differed according to the presence of hyponatremia (*P*<0.001), but the cancerous mortality did not (*P* = 0.482). Subsequently, stepwise multivariate models were applied to calculate the adjusted HRs (Table 2). The patients with hyponatremia had higher HRs of graft survival and mortality than did the counterpart group irrespective of several immunological and non-immunological factors; however, the relationship between hyponatremia and acute rejection remained insignificant after the multivariable-adjustment.

**Table 2:** Hazard ratios for post-transplant outcomes in the hyponatremia group compared with the normonatremia group.

	Model 1		Model 2		Model 3	
Outcome	HR (95% CI)	*P*	HR (95% CI)	*P*	HR (95% CI)	*P*
Biopsy-proven acute rejection	1.39 (0.792–2.424)	0.253	1.33 (0.725–2.429)	0.358	1.38 (0.748–2.552)	0.302
Graft failure	2.99 (1.583–5.645)	0.001	2.96 (1.418–6.176)	0.004	3.21 (1.470–6.994)	0.003
All-cause mortality	5.33 (2.673–10.631)	<0.001	3.10 (1.307–7.357)	0.010	3.03 (1.213–7.542)	0.018

Model 1: unadjusted for covariates.

Model 2: adjusted for age, sex, estimated glomerular filtration rate, diabetes mellitus, history of cardiovascular disease, Hepatitis B virus surface antigen, origin of end-stage renal disease, induction therapy, and other electrolyte findings.

Model 3: adjusted for all covariates.

HR, hazard ratio; CI, confidence interval.

**Fig 3:**
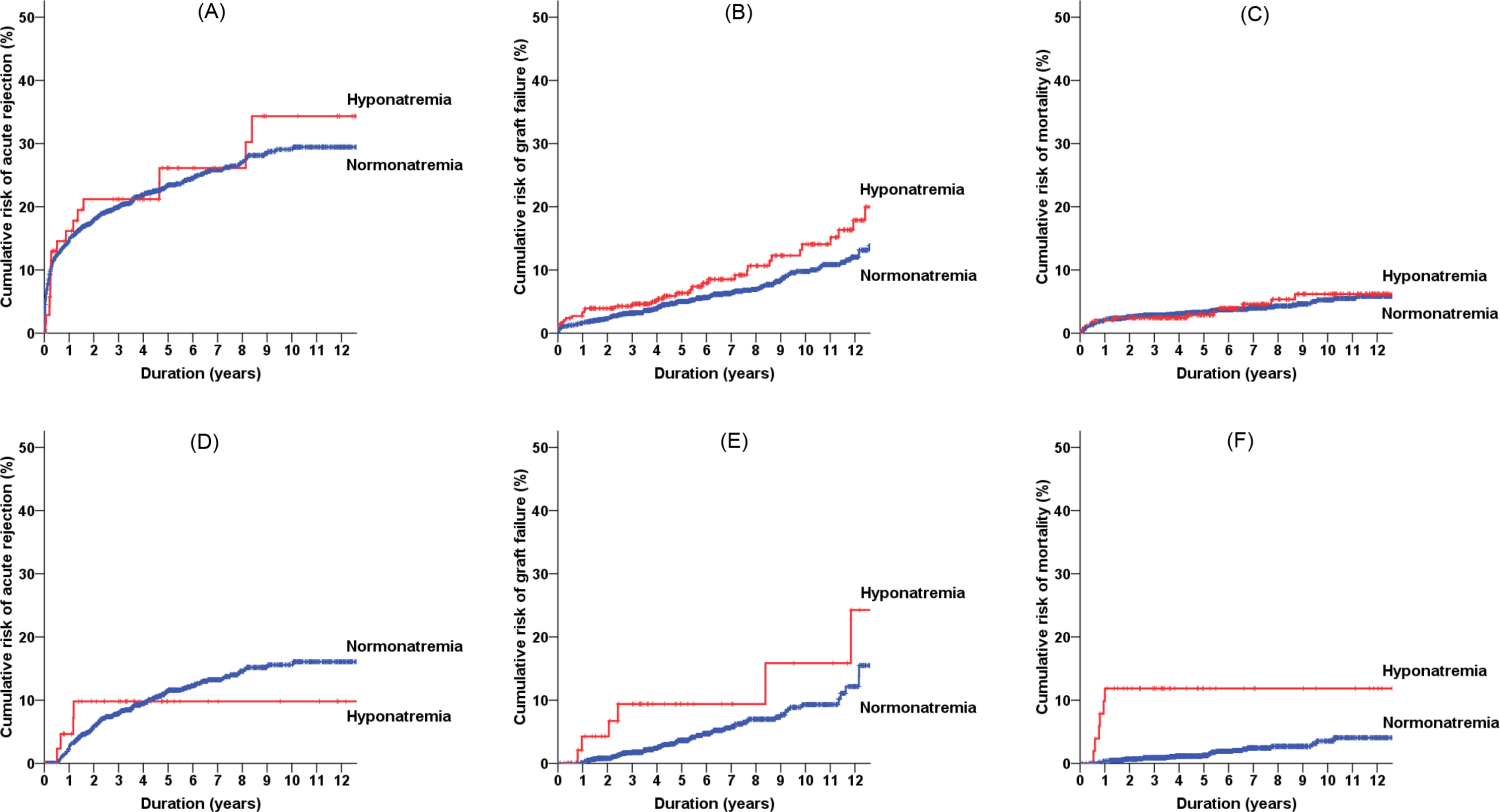
Cumulative risks of acute rejection (A, D), graft failure (B, E), and all-cause mortality (C, F) according to the hyponatremia at baseline (A-C) or 6 months after kidney transplantation (D-F).

We further expanded our hypothesis to the other timeframes, such as baseline (i.e., 0 month) and 6 months after transplantation. However, as stated in the method section, the baseline hyponatremia at the time of kidney transplantation was not associated with any transplant outcomes (Fig 3A–3C). When the hyponatremia was determined at 6 months, the outcome trends were similar to the results obtained from the sodium at 3 months (Fig 3D and 3E).

### Sensitivity analysis

Because heart function is related to the sodium level and to the mortality rate [16], we additionally adjusted for the heart function in the association analysis between the serum sodium and the outcomes. We reviewed the echocardiographic data on the ejection fraction, all of which were examined in the pre-transplant period (n = 1,632). When adjusting for the ejection fraction in the model 3 of Table 2, the HRs for graft failure and mortality remained significant: HR and *P* were 2.82 (1.178–6.762) and 0.020, respectively, for graft failure; HR and *P* were 3.99 (1.450–10.956) and 0.007, respectively, for mortality.

## Discussion

Serum sodium is broadly monitored in current clinical practice because an abnormal level is the most commonly found electrolyte disorder and it can result in poorer outcomes in several clinical settings. With this in mind, the present study has clinical implications on the following issues. First, the present data is the first report on the relationship between serum sodium and kidney transplant outcomes. Second, the relationships were not the same across the outcomes, wherein significance was primarily shown in the overall graft survival and mortality, not in the acute rejection. Finally, the sensitivity analysis (i.e., the adjustment for the ejection fraction) suggests that the relationship mechanisms may not depend on the heart function.

The most common causes of death in kidney transplant recipients are infection and cardiovascular disease [17]. Hyponatremia is related with both infection [18–20] and cardiovascular disease [21], although the issue of the latter is more evident than that of the former. Malignancy, an additional common cause for death, is also known to be related to hyponatremia throughout multiple pathophysiological bases [22]. The present kidney transplant cohort showed a strong significance in the prediction of serum sodium for mortality. With the previous findings in mind, it is plausible that hyponatremia is related to all-cause mortality.

Graft failure is attributable to both immunological and non-immunological factors [3]. The present results showed that hyponatremia was associated with graft loss, but they did not show which immunological and non-immunological processes were underlying this relationship. Intriguingly, there was no correlation between hyponatremia and acute rejection, and the correlation with graft failure was partly dependent on the heart function. With these results in mind, we suggest that serum sodium may affect or be affected more strongly by non-immunological factors than by immunological factors in the context of graft survival or chronic graft insult. However, it should be noted that the hyponatremia may be just a concomitant event for graft outcomes, because the present study could not provide the causal relationship. Accordingly, the future studies are needed to address the causal relationship or its underlying mechanism.

Based on the present data, monitoring post-transplant serum sodium levels may be needed to prepare clinicians for potentially poorer outcomes. However, the issue of hyponatremia correction is a separate matter. First, the current observational study design cannot provide any evidence for the intervention benefit. Second, recent studies have raised a question regarding whether the correction of sodium itself can reduce the mortality risk [9,23]. Hyponatremia may be only a marker of comorbidities, in which the deaths are attributable mainly to the severity of the diseases and not to hyponatremia itself. However, this unsolved question does not hamper the clinical importance of sodium level as a predictor of graft and patient outcomes as shown in the present study.

The present study has strength in the context of the large sample size, the solid statistical significance, and the long-term follow-up duration. Although the data are informative, this study has some limitations. First, as stated above, the study design, which involved observing correlations, limited the drawing of conclusions based on causality. However, the main aim of the present study was to determine the relationship itself; thus, the current design does not significantly hamper this aim. Second, we primarily considered Asians, but not other races; racial disparity in recipients’ state and outcome exists [24]. Third, there were inevitable missing values in certain covariates (e.g., time-varying factors). Nevertheless, we tried to conduct sensitivity analyses for some important factors. Although the present study could not thoroughly solve the latter two limitations, it will form the basis of later studies without these limitations.

For the first time, the present study determined that hyponatremia was associated with poorer outcomes following kidney transplantations. Because current transplant outcomes remain suboptimal, it is essential to find additional factors related to poorer outcomes. In this respect, we believe that monitoring serum sodium and warning clinicians about hyponatremia may improve the transplant and patient outcomes. Future follow-up studies are needed to support the present findings and to address additional issues such as the underlying mechanisms and the correction of hyponatremia in kidney transplant recipients.

## Supporting Information

S1 Data Raw data of the manuscript. (XLS)
